# Translation, cultural adaptation and validation of the English “Short form SF 12v2” into Bengali in rheumatoid arthritis patients

**DOI:** 10.1186/s12955-017-0683-z

**Published:** 2017-05-22

**Authors:** Nazrul Islam, Ikramul Hasan Khan, Nira Ferdous, Johannes J. Rasker

**Affiliations:** 1 Department of Rheumatology, Bangabandhu Sheikh Mujib Medical University, Dhaka, Bangladesh; 2 Modern One stop Arthritis Care and Research Center® (MOAC&RC®), Dhanmondi, Road 8, House 17, Dhaka, Bangladesh; 3 Department of Psychology, Faculty of Behavioural Sciences, Health & Technology, University of Twente, Enschede, The Netherlands

**Keywords:** Short form, SF 12v2, Bangladesh, Bengali, Cross-cultural adaptation, Health-related quality of life

## Abstract

**Background:**

To develop a culturally adapted and validated Bengali **Short Form SF 12v2** among Rheumatoid arthritis (RA) patients.

**Methods:**

The English **SF 12v2** was translated, adapted and back translated into and from Bengali, pre-tested by 60 patients. The Bengali **SF 12v2** was administered twice with 14 days interval to 130 Bangladeshi RA patients. The psychometric properties of the Bengali **SF 12v2** were assessed. Test-retest reliability was assessed by intra-class correlation coefficient (ICC) and Spearman’s rank correlation coefficient and internal consistency by Cronbach’s alpha. Content validity was assessed by index for content validity (ICV) and floor and ceiling effects. To determine convergent and discriminant validity a Bengali Health Assessment Questionnaire (B-HAQ) was used. Factor analysis was done.

**Results:**

The Bengali **SF 12v2** was well accepted by the patients in the pre-test and showed good reliability. Internal consistency for both physical and mental component was satisfactory; Cronbach’s alpha was 0.9. ICC exceeded 0.9 in all domains. Spearman’s rho for all domains exceeded 0.8. The physical health component of Bengali **SF 12v2** had convergent validity to the B-HAQ. Its mental health component had discriminant validity to the B-HAQ. The ICV of content validity was 1 for all items. Factor analysis revealed two factors a physical and a mental component.

**Conclusions:**

The interviewer-administered Bengali **SF 12v2** appears to be an acceptable, reliable, and valid instrument for measuring health-related quality of life in Bengali speaking RA patients. Further evaluation in the general population and in different medical conditions should be done.

**Electronic supplementary material:**

The online version of this article (doi:10.1186/s12955-017-0683-z) contains supplementary material, which is available to authorized users.

## What are new?

_ The Short Form-12v2 (SF12v2) was translated and validated for use in Bengali patients with rheumatoid arthritis.

_ The Bengali SF12v2 administered by interviewers demonstrated psychometric properties similar to the original US English version and translations in other languages.

_ The questionnaire should be evaluated and used in people from the general population and in patients with different medical conditions to assess and compare the health status and impact of different disorders in Bangladeshi patients.

_With about 178 million in Bangladesh and about 261.5 million total speakers worldwide, Bengali it is the sixth most spoken language in the world, so it is important that this questionnaire is now available for studies in this part of the world.

## Background

Measurement of health related quality of life (HRQoL) is increasingly being used in clinical trials and health services research [1, 2]; measuring health related quality is particularly important for measuring the impact of chronic diseases [3]. These HRQoL questionnaires can be divided into generic and specific measures [4, 5]. Generic measures are not specific for any disease or population, and such measures can be used across various diseases. Specific instruments are specific for a disease, a population of patients, ascertain functions, or for a problem [6, 7]. Major advantages of generic instruments include their ability to assess a variety of health domains and to compare HRQoL across different populations, regardless of underlying conditions. Among the generic measures, the Medical outcome study 36-item Short form **(SF 36)** Health survey developed by Ware and Sherburne [8] in the United States is the most widely used [9–12] and has been validated in many countries, including Bangladesh [13–19]. After more than 10 years of experience with the **SF 36** it is reported that it is lengthy, and that some participants may have problems to understand the questions. Then the **SF 12v1** was developed which utilizes only 12 items drawn from each of the eight subscales of the **SF 36** and having the same performance. It was validated for example in nine European countries (Denmark, France, Germany, Italy, the Netherlands, Norway, Spain, Sweden, and the United Kingdom) in Iran and Morocco in the general population [20–23]. More recently, the **SF 12v2** has been described which makes some questions easier than **SF 12v1**. The performance of **SF 12v2** has been reported to be comparable to that of **SF 36** while having the advantage of being easier and quicker to complete [24]. The **SF 12v2** has already been translated and evaluated in many countries [24–27]. Numerous investigators and health care delivery organizations have adopted the **SF 12v2**, including the National Commission on Quality Assurance (NCQA), which chooses the **SF 12v2** for its Annual Member Health Care Survey, and also the Pacific Business Group on Health, which will be one of the first to use it in monitoring outcomes [28]. Questionnaires are the most commonly used techniques for collecting health related information in clinical studies as these are inexpensive, easy and simple to apply and may be used to measure large numbers of health outcomes. There are about 261.5 million Bengali speaking people all around the world and it is the sixth (6th) language according to population [29]. Both at present as well as in the future, there is a need for a valid, reliable and reproducible instrument to compare results and experiences of various therapeutic interventions in such a large population, with different centers. Though the Bengali version of the **SF 36** is available [19] still no cultural adaptation and validation of the **SF 12v2** has been done in Bengali.

Rheumatoid arthritis (RA) is a chronic disabling condition; the functional and the social impact of this disease is enormous. RA can interfere with a person’s ability to function at home, in their job and social situation [30]. Assessment of health status in patients with RA using structured questionnaires has become an important approach to evaluate the treatment and outcome. The Health Assessment Questionnaire (HAQ) has been widely used but highlights the physical problems only [31] whereas the **SF 12v2** assesses the physical as well as the mental component of health. *Bangla* is the language native to the region of Bengal, which comprises the present-day nation of Bangladesh and of the states West Bengal, Tripura in India and southern Assam. It is written using the Bengali alphabet. Bengali is the national language in Bangladesh and second most spoken language in India. With about 178 million native and a total of about 261.5 million speakers worldwide, it is the sixth most spoken language in the world, so it is important to make this questionnaire available for studies in this part of the world.

## Methods

### Original questionnaire

The original SF 12v2 questionnaire contains 12 questions or items. The items are grouped into eight domains measuring physical functioning (PF), role physical (RP, physical health problems), bodily pain (BP), general health (GH), vitality (VT), social functioning (SF), role emotional (RE, emotional problems) and mental health (MH). Higher scores represent better health status [26].

### Translation, cross-cultural adaptation and validation of SF 12 into Bengali

The “forward backward” procedure of Beaton et al. was applied to translate the **SF 12v2** questionnaire [27]. It was carried out in five stages. Two translators whose mother tongue is Bengali have done the forward translation. One of the translators was made aware of the concepts being examined in the questionnaire and the other translator was neither made aware nor informed of these concepts. A synthesized Bengali version was produced which was then back translated into English by two professional translators. Both of them were totally blind of the original version and these two translators without medical background were neither aware nor informed of the concepts being explored in order to avoid information bias and to elicit unexpected meaning of the items in the translated questionnaire.

The expert committee comprised methodologists, health professionals, language professionals, rheumatologists and the translators (forward and back translators). They reviewed and compared all the translations with the original **SF 12v2** questionnaire and verified the semantic, idiomatic, experiential and conceptual equivalence between the English and the Bengali version. Consensus was reached on the items and when necessary the translation and back-translation process was repeated to clarify how another wording of an item can work.

The translation was straightforward for most of the items and response choices except moderate activities, such as moving a table, pushing a vacuum cleaner, bowling or playing golf where the committee added “pushing and or lifting a medium size bucket filled with water as our people do not usually use a vacuum cleaner for dust cleaning.

### Field-testing

The preliminary Bengali version of the SF 12v2 questionnaire was field tested in a random sample of 60 adult RA patients who were enrolled from the outpatient department of Rheumatology of BSMMU. The questionnaire was administered by the investigator in Bengali to each subject who was interviewed. To make sure he would obtain an accurate answer from the respondent the investigator asked what he or she thought was meant by each question item and the chosen response and he or she was encouraged to elucidate his or her understanding of the items and response choice in an open ended manner. This ensured that items and response choice was understood as having a meaning equivalent to that of the source version [28]. The meaning of items was explored. On the basis of this probing the pre final Bengali version was developed in consultation with the expert committee. For example; in question 6 b we put two alternatives “Uddomi or Kormoshakti sampanno” for the word “lot of energy” and used an example for the “emotional problem” in question 7 like “Bishonno or Dushchintagrosto” in for better understanding of people living in different area.

## Psychometric evaluation of the Bengali SF 12v2

### Patients and data collection

A new sample of 130 RA patients was selected. The sample size for this study is determined based on the parameter, the test–retest repeatability. This is measured by the intraclass correlation (r). We expect **SF 12v2** to have an r of 0.8 in this study, and an r of 0.7 or higher would be acceptable to us. Thus, we defined H0: ρ0 = 0.7 and H1: ρ1 = 0.8. Using a two sided test as suggested by Walter et al. [28] with β = 0.2 (80% power) and α = 0.05, a sample size of 117 evaluable subjects would be required. Assuming 10% of subjects might refuse to repeat the **SF 12v2**, a total of 130 subjects would have to be enrolled.

The sample was taken by consecutively inviting RA patients attending the Rheumatology OPD. All patients were adults with RA, > 18 years of age and fulfilling the ACR criteria for RA and were able to understand and co-operate the study procedure. Any patient having a history of co-existing major illness making participation impossible or, psychological illness {considered as exclusion criterion, this group of patients may not communicate well and provided information might produce bias} or unwilling to provide verbal informed consent was excluded from the study. The classification criteria of RA as well as the disease activity in each subject were assessed by a physician informed written consent was taken in front of an attendant. The patients were interviewed by administrating the Bengali SF 12v2 questionnaire. The patients were also asked to indicate whether he or she understood each question, found difficult to answer, thought it irrelevant to him or her, minded answering the question and responses were recorded on the questionnaire. A Bengali version of the Health Assessment Questionnaire B-HAQ was administered separately to each patient and the responses was recorded on the questionnaire.

### Test –retest reliability

Samples of 130 patents were asked to come again after 14 days and were interviewed for the second time. During this interval of 14 days no new intervention was given.

### Scoring of the SF 12v2 questionnaire

The SF 12v2 scales were scored using Likert’s method of summated ratings [29]. This method has been widely used in scale construction because of its simplicity and success in yielding scores. In this method a score for each question item is derived from the algebraic sum of the item score. Score for some of the items needed to be recoded so that all items are scored in the same direction [8]. Raw scores were summated and linearly transformed into 0–100 scale, with 100 indicating the best level, 0 the worst and scores in between representing the percentage of total possible score achieved.

### Statistical analysis

The responses to the Bengali version of SF 12v2 questionnaire were subjected to recommended tests for reliability and validity. The reliability of each scale was assessed through internal consistency. Internal consistency is the extent to which items within a scale (or dimension) are correlated with each other. It was examined by Cronbach’s alpha co-efficient and item scale correlation. Cronbach’s alpha co-efficient is a widely used method which measures the overall correlation between items within a scale. The reliability is considered acceptable when alpha is equal to or exceeds 0.7. Item scale correlation which assesses the extent to which an item is related to the other items of its scale should exceed 0.4. The Reliability was also assessed by the test-retest method. In the retest method the same Bengali version of SF 12v2 questionnaire was given to a sample of patients after 2 weeks and the correlation between the scores on the test and retest were using Spearman correlation co-efficient. The content validity was assessed by the index of content validity (ICV) and assessing floor and ceiling effects by descriptive measures; the floor effect (percentage of patients who scored at floor level – equivalent to the 10% worst results on the scale); and the ceiling effect (percentage of patients who scored at the ceiling level – that corresponded to 10% best results on the scale). The ICV was assessed by three experts: two rheumatologists, one Internist, each expert rated each item as either 1 (agreed), 0 (undetermined), or -1 (disagreed). The ICV of each item was calculated using the summation of scores from each expert divided by the number of experts. Convergent validity was assessed by demonstrating strong correlation between the physical component of the SF12v2 and the Bengali version of the Health Assessment Questionnaire (B-HAQ). Divergent validity was assessed by demonstrating weak correlations between the mental component of the SF12v2 and the B-HAQ.

## Results

### Sociodemographic data

A total of hundred and thirty Bengali speaking RA patients agreed to participate in this study after explanation of the nature of the study. One hundred and nineteen subjects completed the 2^nd^ visit. There were 92 (77%) female and 27 (22.7%) male patients. The mean age of the participants were 36.43 (SD 9.47) with age range from 18 to 60 years. Seventy five (63%) participants were RA factor positive. The mean disease duration of the participants was 6.42 (SD 5.84) years which ranged from 6 months to 15 years.

### Acceptability

All the 130 participants answered all the questions of Bengali version of the SF 12v2. There were no patients who did not understand the questions. Very few patients had difficulty in understanding some items: MH 5 (3.84%) and VT 11(8.46%). Some patients 8 (6.15%) said that social functioning is not relevant to them (Table 1). The main reason given was they had very few social activities. Nobody minded answering the questions.

**Table 1:** Number (%) of the patents who had problems in Bengali SF 12v2

Domain	Do not understand	Difficult to understand	Not relevant	Mind answering
General health	0	0	0	0
Physical functioning	0	0	0	0
Role-physical	0	0	0	0
Role emotional	0	0	0	0
Bodily pain	0	0	0	0
Mental health	0	5 (3.84%)	0	0
Vitality	0	11(8.46%)	0	0
Social functioning	0	0	8 (6.15%)	0

### Response distribution

All values were observed for each domain. The RA patients showed restriction in their physical health in all domains (GH, PF, RP and BP). The items within these domains scored at lower levels on 0–100 scale. Mental health domains (RE, MH, VT and SF) were scored at higher levels. Among the Mental health domains SF were scored (72.9) at a highest level, a reflection of maintenance of social life with disease.

### Distribution of the SF 12v2 scores

The mean domain scores ranged from 26.6 (GH) to 72.90 (SF). The RE, MH, VT and SF domains were slightly skewed to the left whereas the GH, PF, RP and BP slightly skewed to the right. The percentage of patients scoring at the lowest level was pronounced in GH (20.2%), RE (21.8%) and BP (27.7%). A ceiling effect was seen for PF (32.8%) and SF (40.3%) (Table 2).

**Table 2:** Descriptive statistics of the Bengali SF 12v2 scores

Domain	Range	Mean	SD	Skew ness	Floor effect^a^ (%)	Ceiling effect^b^ (%)
GH	0–75	26.26	17.78	0.36	20.2	2.5
PF	0–100	41.70	36.87	0.34	20	32.8
RP	0–100	43.95	27.93	0.40	10.1	7.6
RE	25–100	62.18	26.21	-0.06	21.8	17.6
BP	0–100	43.70	35.09	0.12	27.7	12.6
MH	0–100	57.04	27.50	-0.14	2.5	7.6
VT	0–100	55.67	31.80	-0.24	10.1	16.8
SF	0–100	72.90	29.93	-0.98	5	40.3

*SD* standard deviation, *GH* general health, *PF* physical functioning, *RP* role-physical, *RE* role emotional, *BP* bodily pain, *MH* mental health, *VT* vitality, *SF* social functioning

^a^Percentage of respondents with worst possible score

^b^Percentage of respondents with best possible score

### Reliability

**Internal consistency:** was assessed using Cronbach’s alpha. The results showed that the alpha exceeded the 0.70 for both summary measures level. The alpha for physical component summary (PCS) was 0.906 and mental component summary (MCS) was 0.908 (Table 3).

**Table 3:** Reliability of Bengali SF 12v2 (*n* = 119)

SF 12v2 summery	Domain	Test retest reliability			Cronbach’s alpha
		Spearman’s correlation	ICC	95% CI	
PCS	General health	1	0.992	(.989, .994)	
	Physical functioning	0.999	0.996	(.994, .997)	
	Role-physical				0.906
	Bodily pain	0.993	0.991	(.988, .994)	
	Role-emotional	0.995	0.994	(.991, .996)	
MCS	Mental health				
	Vitality	0.995	0.995	(.993, .997)	
	Social functioning	0.997	0.998	(.998, .999)	0.908
		0.997	0.997	(.996, .998)	
		0.992	0.994	(.992, .996)	

Note: *PCS* physical component summery, *MCS* mental component summary, *ICC* intraclass correlation coefficient

**Test- retest reliability:** was done in 119 patients. Spearman’s rank correlation coefficient for the result of the test and retest in all domains was above 0.9. It ranged from 0.992 to 0.999. For GH it was highest 1 (Table 3).

The intraclass correlation coefficient (ICC) for all domains was above 0.8. The highest was 0.998 for MH and the lowest was 0.991 for RP (Table 3).

### Validity

#### Content validity

The ICV (index for content validity) was 1 for all items in the domains. The Bengali version of SF 12v2 had acceptable ceiling effect in all domains except PF (32.8%) and SF (40.3%). Floor effects were also acceptable except RE (21.7%) and BP (27.7%).

### Construct validity

#### Factor analysis

By principal component analysis two underlying factors were identified, one representing the “physical” aspect of health and one representing the “mental” aspect of health which together explained 80% of the total variance. As presented in the Table 4 the physical domains (GH, PF, RP, and BP) had higher correlations (range 0.80 to 0.84) with the physical component then with the mental component (range 0.10 to 0.42). The mental domain (RE, MH, VT and SF) showed the opposite pattern; range of correlation with the physical component (0.12 to 0.51) and range of correlation with mental component (0.78 to 0.88) (Table 4).

**Table 4:** Factor analysis for the factors of Bengali SF 12v2

Domain	Component	
	1 (Physical)	2 (Mental)
General health	0.84541	0.106328
Physical functioning	0.827674	0.287226
Role-physical	0.809868	0.422559
Role emotional	0.125812	0.84526
Bodily pain	0.800717	0.424837
Mental health	0.30602	0.886262
Vitality	0.511844	0.784211
Social functioning	0.295419	0.792428

### Internal construct validity

The correlation between the domains ranged from 0.28 (between GH and SF) to 0.65 (between GH and BP), well below the preset 0.70 level of distinctiveness of the concept being measured. All the domains had higher correlations with themselves. Stated in another way, the inter domain correlations were less than that of the internal domain correlation, showing that each domain measured a unique concept to other domains. Generally higher correlations were found between the domains of the same dimension (physical or mental) and lower correlations were found between different dimensions (Table 5).

**Table 5:** Spearman’s correlations between the domains of Bengali SF 12v2

Correlation between domains								
Scales	General health	Physical functioning	Role physical	Role emotional	Bodily pain	Mental health	Vitality	Social functioning
General health	1							
Physical functioning	0.60	1						
Role-physical	0.64	0.77	1					
Role emotional	0.29	0.38	0.5	1				
Bodily pain	0.65	0.68	0.86	0.38	1			
Mental health	0.43	0.49	0.54	0.74	0.6	1		
Vitality	0.54	0.6	0.68	0.64	0.78	0.9	1	
Social functioning	0.28	0.49	0.64	0.57	0.6	0.70	0.71	1

### Convergent and discriminant validity

Scores for the SF 12v2 and B-HAQ had shown acceptable convergent and discriminant validity. Strong correlations were found between the domains of physical concept of SF 12v2 and B-HAQ. The highest correlation was found between the B-HAQ and SF 12v2 (PF) -0.82 and the lowest correlation found between B-HAQ and SF 12v2 (RE) -0.35 (Table 6).

**Table 6:** Convergent and discriminant validity of Bengali SF 12v2

	B.HAQ	General health	Physical functioning	Role physical	Role emotional	Bodily pain	Mental health	Vitality	Social functioning
B.HAQ	1								
GH	-0.717	1							
PF	-0.813	0.601	1						
RP	-0.821	0.643	0.771	1					
RE	-0.356	0.293	0.378	0.502	1				
BP	-0.801	0.646	0.681	0.863	0.376	1			
MH	-0.509	0.426	0.49	0.538	0.741	0.601	1		
VT	-0.669	0.544	0.602	0.677	0.636	0.783	0.904	1	
SF	-0.484	0.277	0.49	0.641	0.568	0.6	0.703	0.71	1

*B.HAQ* Bengali Health Assessment Questionnaire, *GH* general health, *PF* physical functioning, *RP* role-physical, *RE* role emotional, *BP* bodily pain, *MH* mental health, *VT* vitality, *SF* social functioning

## Discussion

In this study, the Bengali version of SF 12v2 demonstrated acceptable psychometric properties in terms of reliability and validity in Bengali speaking adult RA patients. Thus the SF 12v2 appears to be a reliable and valid measure of HRQoL in RA patients in this socio-cultural context. The SF12v2 was validated in other countries [7–24] and this is the first study validating the SF 12v2 in Bangladesh. Even though the guidelines for translation were carefully structured and followed, compliance with them did not ensure that the translation was adequate in every aspect, which may be due to the magnitude of linguistic and cultural differences between Bangladesh and the United States. However all the modified questions were understood by all the respondents and these questions were adapted in the prefinal version of Bengali SF 12v2. We found the following tactics as used by other previous researchers [31] to be crucial in minimizing linguistic and cultural difficulties and in overcoming those that did arise.

i) The translators were asked to provide all reasonable options before the group meeting. This encouraged them to think thoroughly about problematic questions items and response choice, which resulted in material for productive discussion and eventually consensus, during the group meeting.
ii) The concepts expressed by all problematic expressions and words in the English version were discussed thoroughly. This was particularly important because if a translator misunderstood a concept, the result was often an overly literal Bengali translation.
iii) A survey in the pilot study was useful for testing minor revisions, and the suggestions of experts in languages were helpful when question items and response choice were found to be unacceptable despite consensus of the translation team.
iv) Expert committee discussion: Discussion in the expert committee meeting provided valuable guiding when our review of the pilot study indicated that the preliminary Bengali version of SF 12v2 had some problems. Expert group discussions were used to identify the problems and to a develop solution, that is a better translation. Thus, the procedure of translation, back translation, field testing and adaptation included frequent evaluation.

Overall, the questionnaire’s performance, regarding convergent and discriminant validity was found to be very encouraging. The correlation between domains indicates that the eight domains are internally consistent. The high success rate on the test of discriminant validity indicates that each of the eight domains can be used to measure different aspect of health status. The principal component analysis revealed two components that support the existence of the two hypothesized dimensions underlying the SF 12v2, the physical and mental health component. Taken together the two factors explained 80% of the total variance. The correlations pattern between SF 12v2 scales and the rotated components revealed the higher correlation of physical domains (Physical functioning, Role physical, Bodily pain, General health) with the first factor. The Mental health domains correlate weakly with this factor. In accordance with the observed pattern, the first factor was labeled as physical health and the second as mental health as hypothesized in the original English version of SF 12v2.

This study also provided empirical evidence for the reliability of the Bengali SF 12v2. The reliability of all domains was well above the 0.70 standard for group comparison. The internal consistency of the Bengali SF 12v2 was shown by Cronbach’s alpha of >0.9 to be similar to that reported in other studies where alpha ranges from 0.78 to 0.95. The test retest reliability of the Bengali SF 12v2 was comparable with previous reports in Korean and Iran where correlation ranged from 0.87 to 0.99 [24, 32]. The Bengali version of SF 12v2 had shown consistency and feasibility when served in RA patients in this series. The validated Bengali SF 12v2 is incorporated in this article as an Additional file 1 for wider use of this questionnaire by Bengali speaking people.

Although, patients found the HAQ easier to complete, the HAQ is limited in providing information on functional health and pain whereas as the SF 12v2 provides a broader picture of health including emotional status role functioning and anxiety and depression.

Validation of a health survey does not end with a single study, but continues with repeated use of an instrument. Future studies of this Bengali version of SF 12v2 should evaluate how well this instrument discriminates among groups differing in diagnosis, disease severity and treatment responses. Its validity in predicting future health and utilization of health services should also be tested. The more frequently an instrument is used in the more situations in which it performs as expected the greater will be our confidence in its validity.

## Conclusions

- The translated and culturally adapted Bengali version of SF 12v2 is an acceptable, reliable and valid instrument that can be administered to Bangladeshi patients with Rheumatoid arthritis for evaluation of their function and disability.
- The SF 12vs2 is an instrument to measure generic and specific health related quality of life (HRQoL) in a quick and easy way and it is increasingly being used in clinical trials and health services research.
- We completed the first Bengali version of the questionnaire.
- The Bengali SF12v2 administered by interviewers demonstrated psychometric properties similar to the original US English version and translations in other languages.
- With about 178 million in Bangladesh and about 261.5 million total speakers worldwide, Bengali it is the sixth most spoken language in the world, so it is important that this questionnaire is now available for studies in this parts of India and Bangladesh.
- The questionnaire should be evaluated and used in people from the general population and in patients with different medical conditions to assess and compare the health status and impact of different disorders in Bengali speaking patients.

## Additional file

Additional file 1: Bengali SF 12v2 Health Survey. (PDF 189 kb)

## Abbreviations

B-HAQ: Bengali Health Assessment Questionnaire
BMMSU: Bangabandhu Sheikh Mujib Medical University
BP: Bodily pain
GH: General health
HAQ: Health Assessment Questionnaire
HRQoL: Health related quality of life
ICC: Intraclass correlation coefficient
ICV: Index for content validity (ICV)
MCS: Mental component summary
MH: Mental health
NCQA: National Commission on Quality Assurance
PCS: Physical component summary
PF: Physical functioning
RE: Role emotional (emotional problems)
RP: Role-physical (physical health problems)
SD: Standard deviation
SF: Social functioning
SF12v2: Short form-12 version 2
VT: Vitality
