# Supplementary material for: Translation, cultural adaptation and validation of the English “Short form SF 12v2” into Bengali in rheumatoid arthritis patients
Source: Health Qual Life Outcomes. 2017 May 22;15:109. doi: 10.1186/s12955-017-0683-z (PMC5441088; doi:10.1186/s12955-017-0683-z)
Supplement: Additional file 1: — Bengali SF 12v2 Health Survey. (PDF 189 kb) [file 12955_2017_683_MOESM1_ESM.pdf]

Bengali SF 12v2 Health Survey

এই জরিপে আপনার স্বাস্থ্য সম্পর্কে আপনার দৃষ্টিভঙ্গি জানতে চাওয়া হয়েছে। আপনি কেমন বোধ করেন এবং আপনার স্বাভাবিক কার্যক্রম কতটা ভালোভাবে করতে পারেন এই জরিপ তা বুঝতে সাহায্য করবে।

নিচের প্রত্যেকটি প্রশ্নের জন্য একটি করে টিক চিহ্ন দিন, যেটি আপনার জন্য সবচেয়ে সঠিক উত্তর বলে আপনি মনে করেন।

১) সব মিলিয়ে আপনি কি বলবেন আপনার স্বাস্থ্য কেমন?

○ চমৎকার ○ খুব ভালো ○ ভালো ○ মোটামুটি ○ খারাপ

২) নিচের প্রশ্নগুলো আপনি প্রতিদিন যে কাজগুলো করতে পারেন সে সম্পর্কিত । আপনার স্বাস্থ্য কি বর্তমানে সেসব কর্মকাণ্ডে ব্যাঘাত ঘটায় ? ঘটালে কতটুকু?

ক) মাঝারি কাজ যেমনঃ একটি টেবিল সরানো, মাঝারি সাইজের পানি ভর্তি একটি বালতি সরানো, ভ্যাকুয়াম ক্লিনার ঠেলা, বোলিং বা গলফ খেলা

○ হ্যাঁ, অনেকটা বাধাগ্রস্ত করে

○ হ্যাঁ, কিছু বাধাগ্রস্ত করে

○ না, মোটেই বাধাগ্রস্ত করে না

খ) বেশ কয়েক ধাপ সিঁড়ি বেয়ে ওঠা

○ হ্যাঁ, অনেকটা বাধাগ্রস্ত করে

○ হ্যাঁ, কিছু বাধাগ্রস্ত করে

○ না, মোটেই বাধাগ্রস্ত করে না

৩) গত চার সপ্তাহে শারীরিক কারণে আপনার কাজ বা অন্যান্য দৈনন্দিন কর্মকাণ্ডের জন্য নিচের সমস্যাগুলো কত সময় ধরে হয়েছিল?

ক) যতটা কাজ করতে চেয়েছিলেন তার চেয়ে কম করতে পেরেছেন

0 সবসময় 0 বেশিরভাগ সময় 0 কিছু সময় 0 খুব অল্প সময় 0 কখনই না।

খ) কোনো কোনো কাজে সীমাবদ্ধতা ছিল

0 সবসময় 0 বেশিরভাগ সময় 0 কিছু সময় 0 খুব অল্প সময় 0 কখনই না।

৪) গত চার সপ্তাহে আবেগজনিত সমস্যা, যেমনঃ বিষণ্ণ বা দুশ্চিন্তাগ্রস্থ থাকার কারণে আপনার কাজ বা অন্যান্য দৈনন্দিন কর্মকাণ্ডের জন্য নিচের সমস্যাগুলো কত সময় ধরে হয়েছিল?

ক) যতটা কাজ করতে চেয়েছিলেন তার চেয়ে কম করতে পেরেছেন

0 সবসময় 0 বেশিরভাগ সময় 0 কিছু সময় 0 খুব অল্প সময় 0 কখনই না।

খ) যেভাবে মনোযোগের সাথে কাজগুলো করেন তারচেয়ে কম মনোযোগী ছিলেন

0 সবসময় 0 বেশিরভাগ সময় 0 কিছু সময় 0 খুব অল্প সময় 0 কখনই না।

৫) গত চার সপ্তাহে ব্যথাজনিত কারণে ঘরের এবং ঘরের বাইরের স্বাভাবিক কাজগুলো করতে আপনার কতটা সমস্যা হয়েছিল ?

0 একেবারেই না 0 খুব অল্প 0 মোটামুটি 0 অনেকটা 0 খুব বেশী

৬) গত চার সপ্তাহে আপনার সার্বিক অবস্থা কেমন ছিল এবং আপনি কেমন বোধ করেছিলেন, এই প্রশ্নগুলো সে সম্পর্কিত।

প্রত্যেকটি প্রশ্নের জন্য অনুগ্রহ করে একটি উত্তর নির্ধারণ করুন যা আপনার অনুভূতির সবচেয়ে কাছাকাছি ।

ক) গত চার সপ্তাহের কতটা সময় আপনি স্থির এবং শান্তিপূর্ণ অনুভব করেছিলেন?

0 সবসময় 0 বেশিরভাগ সময় 0 কিছু সময় 0 খুব অল্প সময় 0 কখনই না।

খ) আপনি কি অনেক উদ্যোমী বা কর্মশক্তিসম্পন্ন ছিলেন?

0 সবসময় 0 বেশিরভাগ সময় 0 কিছু সময় 0 খুব অল্প সময় 0 কখনই না।

গ) আপনি কি মনমরা এবং হতাশ বোধ করেছিলেন?

0 সবসময় 0 বেশিরভাগ সময় 0 কিছু সময় 0 খুব অল্প সময় 0 কখনই না।

৭) শারীরিক এবং আবেগজনিত সমস্যা, যেমনঃ বিষণ্ণ বা দুশ্চিন্তাগ্রস্থ থাকার কারনে গত চার সপ্তাহের কতটা সময় সামাজিক কর্মকাণ্ড (যেমনঃ বন্ধুবান্ধব বা আত্মীয়স্বজন ইত্যাদির সাথে দেখা করা) বিঘ্নিত হয়েছে ?

0 সবসময় 0 বেশিরভাগ সময় 0 কিছু সময় 0 খুব অল্প সময় 0 কখনই না।

প্রশ্নগুলো পূরণ করার জন্য ধন্যবাদ।
